# Two-staged surgical repair of Berry syndrome type 2B

**DOI:** 10.1093/icvts/ivac284

**Published:** 2023-01-09

**Authors:** Connor J Byeman, Krista Young, Ravi Ashwath

**Affiliations:** Division of Pediatric Cardiology, Department of Pediatrics, Stead Family Children’s Hospital, University of Iowa Hospitals and Clinics, Iowa City, IA, USA; Division of Pediatric Cardiology, Department of Pediatrics, Stead Family Children’s Hospital, University of Iowa Hospitals and Clinics, Iowa City, IA, USA; Division of Pediatric Cardiology, Department of Pediatrics, Stead Family Children’s Hospital, University of Iowa Hospitals and Clinics, Iowa City, IA, USA

**Keywords:** Berry syndrome, Interrupted aortic arch, Aortopulmonary window, Patent ductus arteriosus, Coarctation of the aorta, Pulmonary artery stenosis

## Abstract

Berry syndrome is a rare congenital heart disease that requires complete corrective surgery. In certain extreme cases, such as ours, a two-stage as opposed to single-stage repair is a possibility. In doing so, we also used annotated and segmented three-dimensional models for the first time in Berry syndrome, adding to growing evidence that such models enhance the understanding of complex anatomy for surgical planning.

## INTRODUCTION

Berry syndrome is an extremely rare congenital heart disease complex involving an aortopulmonary window, a right pulmonary artery (PA) that arises from the ascending aorta, an interrupted aortic arch with a patent ductus arteriosus and an intact ventricular septum. The usual management is a single-stage corrective surgery.

## CASE REPORT

**Figure 1:**
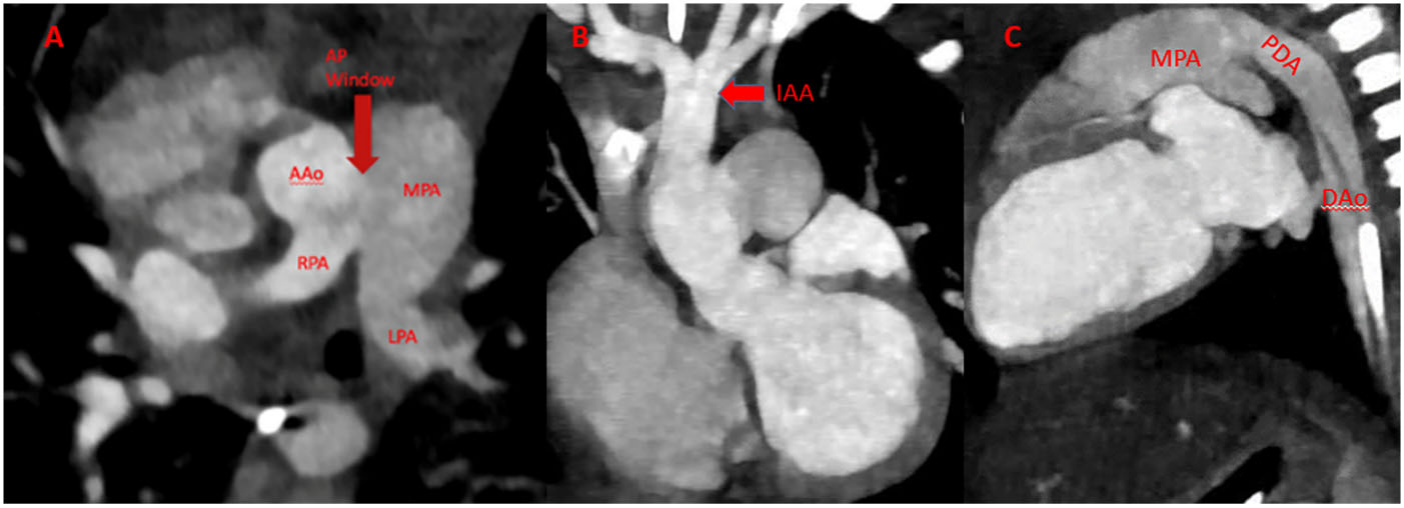
Computed tomographic images demonstrating: (**A**) a type III aortopulmonary window (AP window) as well as the aortic origin of the right pulmonary artery (RPA) from the ascending aorta (AAo), (**B**) the type A interruption of the aortic arch with no visible continuation after the third aortic branch to the descending aorta and (**C**) the patent ductus arteriosus (PDA) connecting the main pulmonary artery (MPA) to the descending aorta (DAo).

**Figure 2:**
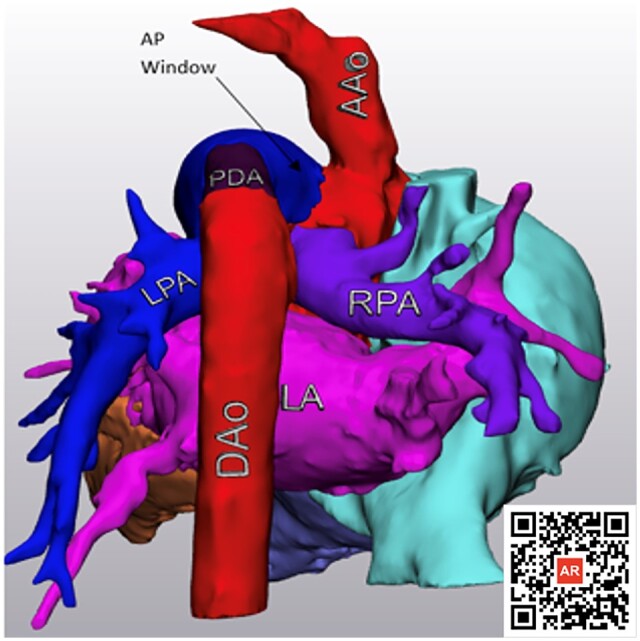
A posterior view of the heart, modelled by segmentation software. A scannable AR code is provided to allow access anytime to the entire model. This image shows the extent and borders of the AP window (indicated by an arrow), interrupted aortic arch and aortic origin of the right pulmonary artery. AAo: ascending aorta; AP window: aortopulmonary window; AR: augmented reality; DAo: descending aorta; LPA: left pulmonary artery; PDA: patent ductus arteriosus; RPA: right pulmonary artery.

A full-term 2.6-kg male neonate with suboptimal prenatal care required intubation secondary to respiratory distress 6 h after delivery. There were decreased lower extremity pulses on exam. A transthoracic echocardiogram demonstrated a type III aortopulmonary window, critical coarctation of the aorta, a right PA origin from the aorta, an intact ventricular septum and a patent ductus arteriosus (PDA). Prostaglandins were started for duct maintenance. Due to the complexity of the anatomical arrangement, a computed tomographic (CT) scan was obtained to better delineate the anatomy. The images confirmed the findings of the echocardiogram and additionally demonstrated type A interruption of the aorta. This constellation of findings led to the diagnosis of Berry syndrome type 2B (Fig. 1). An exact three-dimensional (3-D) reconstruction of the anatomy created via the CT angiogram is shown below, alongside a scannable augmented reality code for at-home access of the complete model (Fig. 2).

Despite optimization of medical management with ventilation, prostaglandins, diuretics, inotropes and sedation with paralysis, the patient had worsening unbridled pulmonary overcirculation and low systemic blood pressure. A hybrid procedure consisting of bilateral PA banding and PDA stenting was performed on day 6 of life. The patient was deemed too unstable for a complete repair at that time. On day 15 of life, complete repair consisting of en-bloc excision of the right PA with reattachment to the main PA posteriorly through the aortopulmonary window, closure of the aortopulmonary window, aortic arch reconstruction augmented with homograft patch material and removal of the PA bands and PDA with stent was performed. Antegrade cerebral perfusion was used to maintain cerebral pressure throughout cardiopulmonary bypass. The postoperative course was uneventful. During a 9-month follow-up visit, routine echocardiography showed significant right PA stenosis, which required balloon angioplasty. At 18 months of age, the patient is doing well with no cardiovascular concerns.

## DISCUSSION

Berry syndrome is an extremely rare congenital cardiac defect that was first characterized in 1982 by Berry *et al.* [1]. These patients typically present with moderate to severe respiratory distress and do not usually have associated visceral malformations. As has been discussed previously, cross-sectional imaging modalities like CT angiography can be used to precisely diagnose the defect and define the anatomy prior to surgery [2]. With recent advances in 3-D modelling, we generated a 3-D PDF and used an augmented reality code to depict this case’s intricate anatomy in 3-D space. This proved to be a great resource to our care team helping with diagnosis and surgical planning of the second stage repair in this rare congenital cardiac abnormality. It was felt that this model was the most helpful imaging modality, despite advanced CT technology.

Typically, treatment for Berry syndrome is a one-stage complete repair of the defect [3]. The novelty of this case lies with the decision and feasibility to perform a staged repair. Our patient presented in extremis with severe pulmonary overcirculation and compromised systemic perfusion. Thus, the patient was too unstable for single-stage complete repair. After discussion and review of his multimodality imaging, both cardiology and the surgical team collaboratively decided to proceed with a staged repair, starting initially with a hybrid procedure to allow time for the patient to be clinically optimized prior to complete repair. This has been previously reported only once [4]. However, in that case, the initial procedure was limited to PA banding and continuation of prostaglandins to maintain duct patency. In both situations, the need for stabilization of the patient prior to repair was paramount.

## CONCLUSION

As evidenced by this case, a two-stage repair could be an option when patients with Berry syndrome present in a condition that prevents typical one-stage repair. Further data will need to be collected regarding long-term surgical outcomes in cases such as this. Annotated and segmented 3-D models can enhance the understanding of complex anatomy for surgical planning.

## PERMISSION

Permission for authorship and publication of this article was provided by the parents of the patient.

## Data Availability

No new data were generated.
